# Supplementary material for: Coordination Polymers Bearing Angular 4,4′-Oxybis[N-(pyridin-3-ylmethyl)benzamide] and Isomeric Dicarboxylate Ligands: Synthesis, Structures and Properties
Source: Molecules. 2025 Aug 5;30(15):3283. doi: 10.3390/molecules30153283 (PMC12348699; doi:10.3390/molecules30153283)

Supplementary Materials

Coordination Polymers Bearing Angular 4,4′-Oxybis[*N*-(pyridin-3-ylmethyl)benzamide] and Isomeric Dicarboxylate Ligands: Synthesis, Structures and Properties

Yung-Hao Huang [1], Yi-Ju Hsieh [1], Yen-Hsin Chen [1], Shih-Miao Liu [2,*] and Jhy-Der Chen [1,*]

[1] Department of Chemistry, Chung Yuan Christian University, Chung Li, Taoyuan City 320314, Taiwan; a0987198719@gmail.com (Y.-H.H.); yovr2840@gmail.com (Y.-J.H.); sunny911228@gmail.com (Y.-H.C.)

[2] Center for General Education, Hsin Sheng Junior College of Medical Care and Management,
Longtan 32544, Taiwan

* Correspondence: lsm0301@hsc.edu.tw (S.-M.L.); jdchen@cycu.edu.tw (J.-D.C.); Tel.: +886-3-265-3351 (J.-D.C.)

Figure S1. (a) Simulated and (b) experimental PXRD patterns of complex **1**.

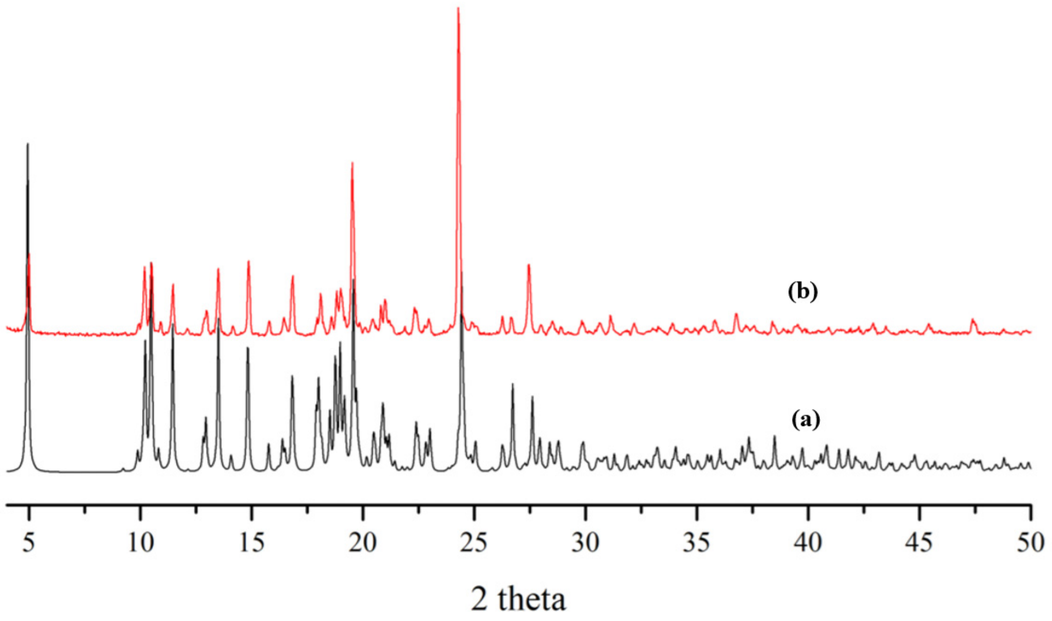

Figure S2. (a) Simulated and (b) experimental PXRD patterns of complex **2**.

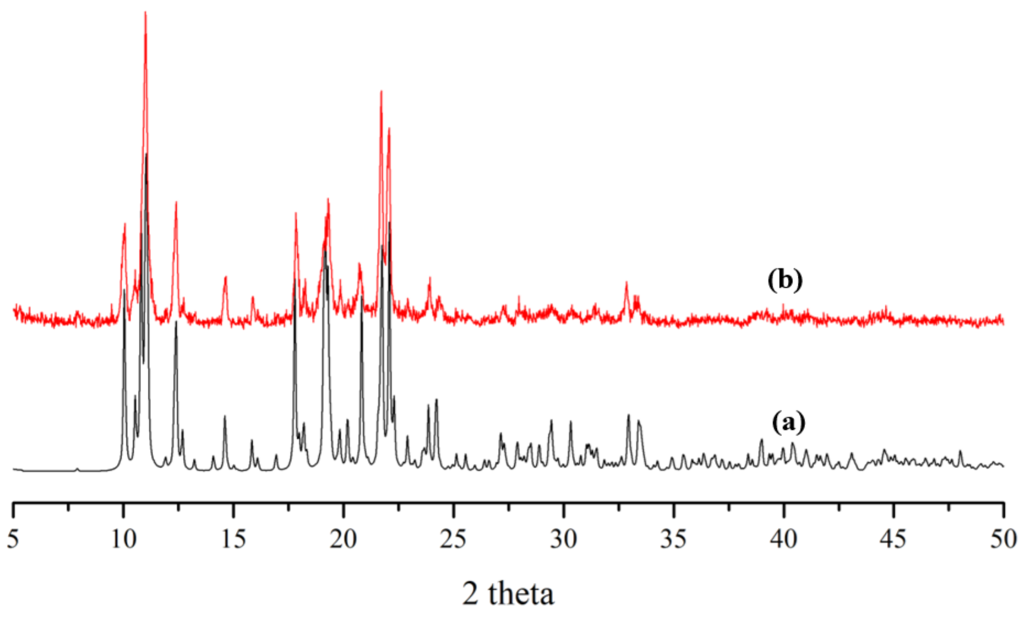

Figure S3. (a) Simulated and (b) experimental PXRD patterns of complex **3**.

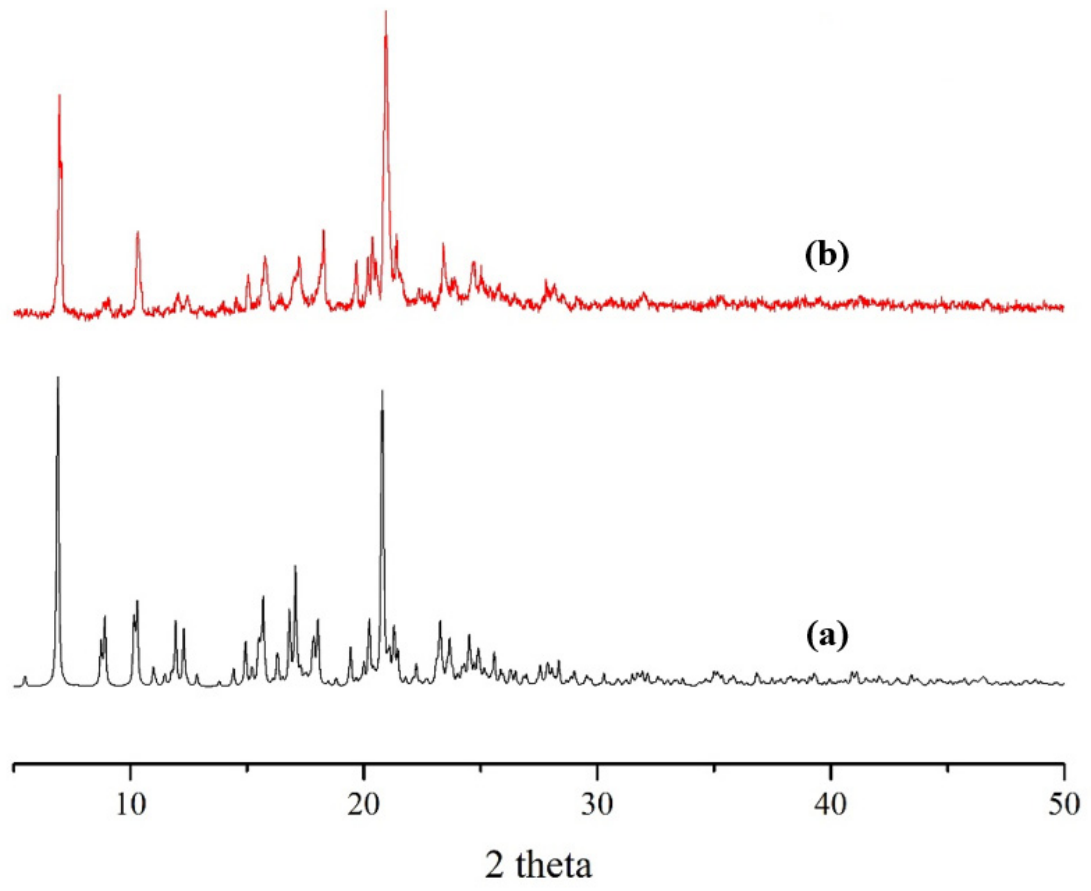

Figure S4. (a) Simulated and (b) experimental PXRD patterns of complex **4**.

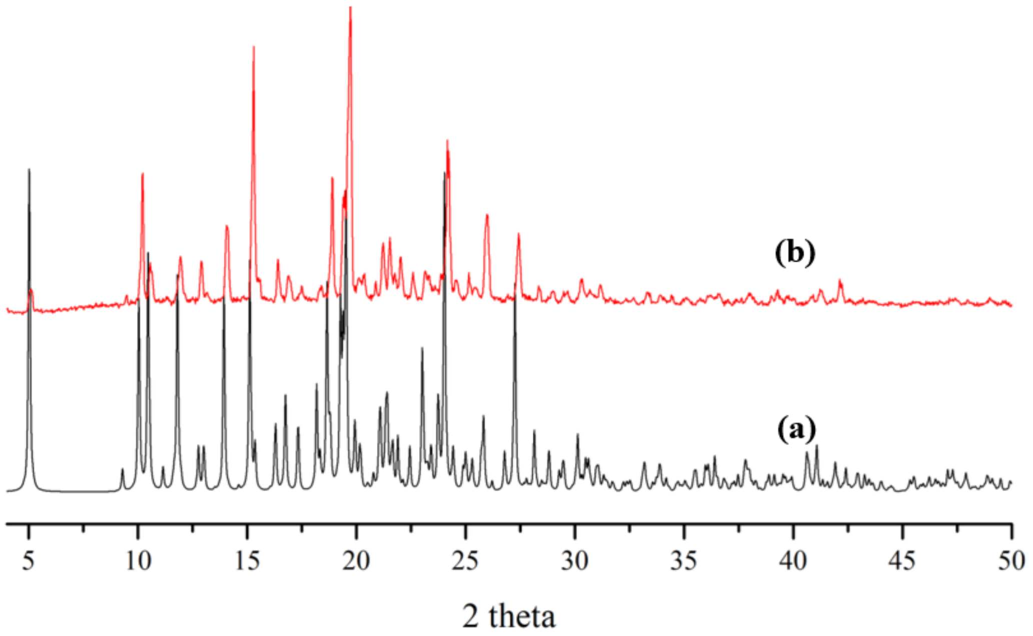

Figure S5. (a) Simulated and (b) experimental PXRD patterns of complex **5**.

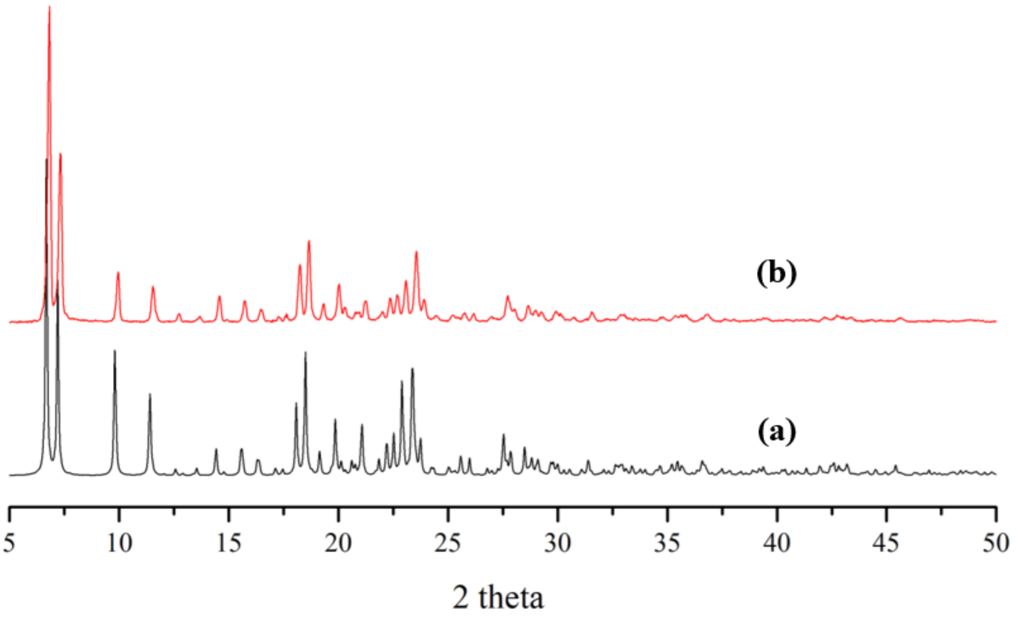

Figure S6. (a) Simulated and (b) experimental PXRD patterns of complex **6**.

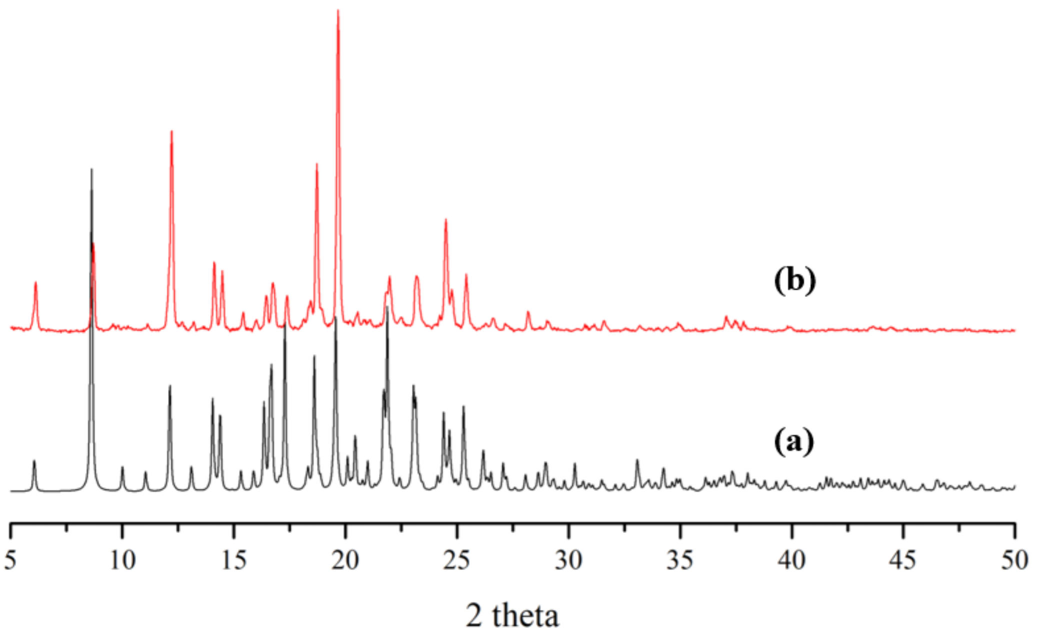

Figure S7. (a) Simulated and (b) experimental PXRD patterns of complex **7**.

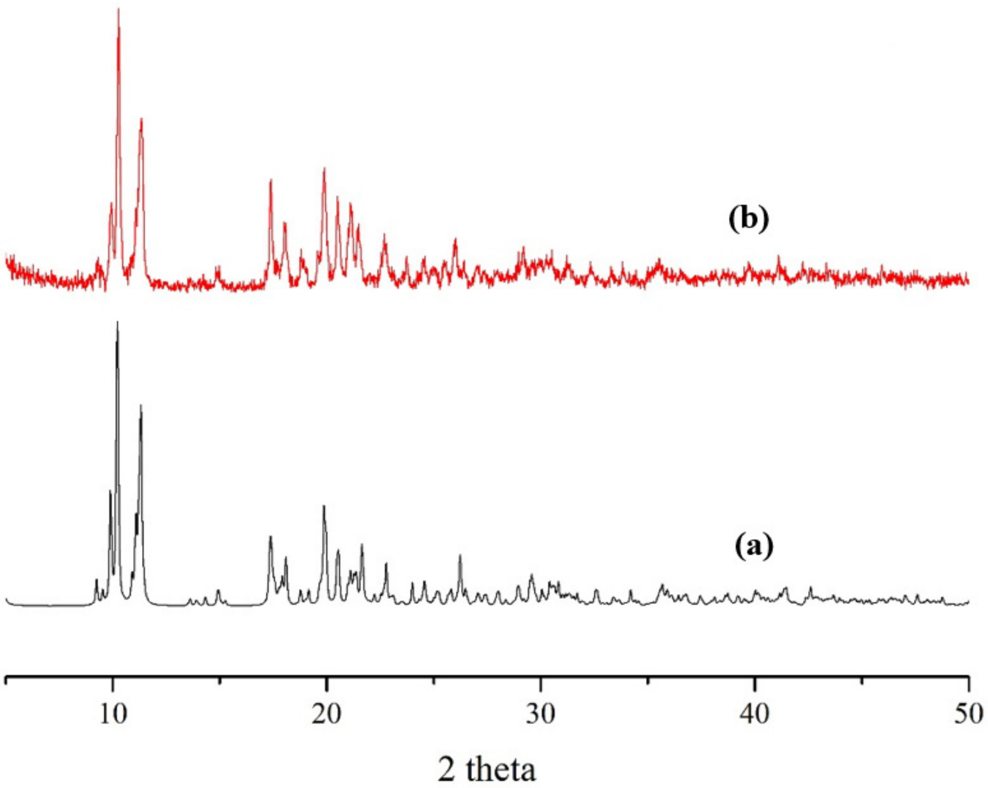

Figure S8. TGA curve of complex **1**.

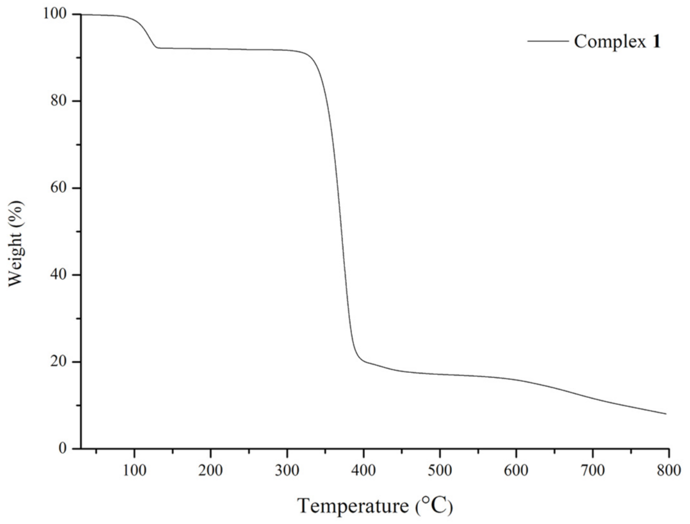

Figure S9. TGA curve of complex **2**.

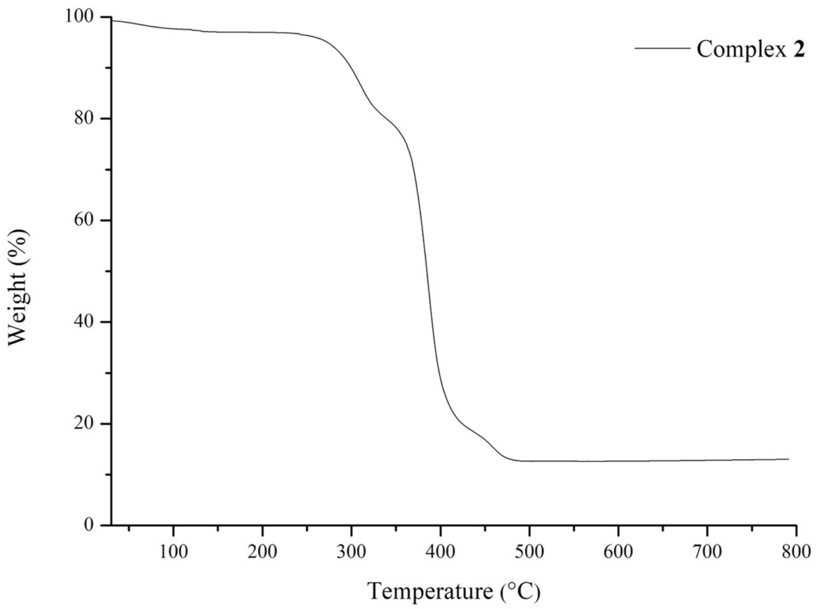

Figure S10. TGA curve of complex **3**.

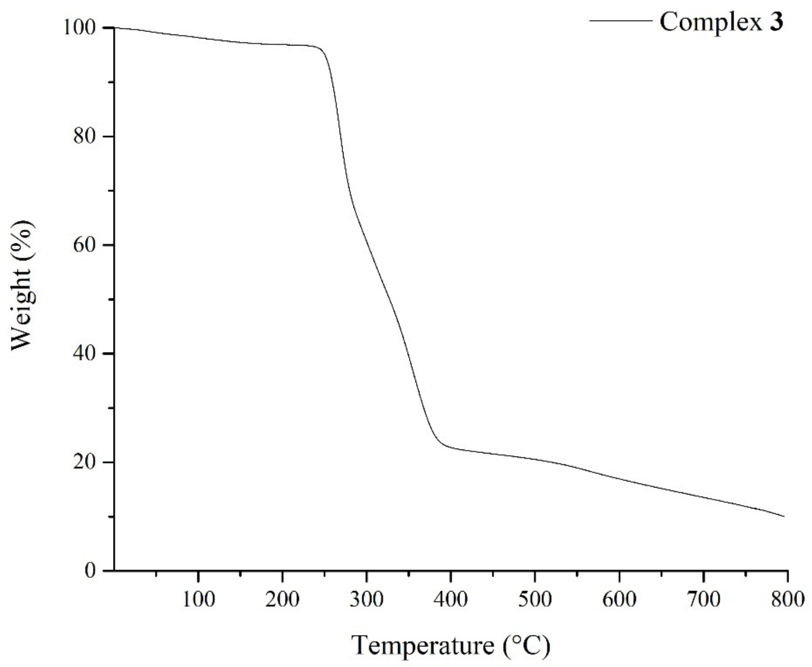

Figure S11. TGA curve of complex **4**.

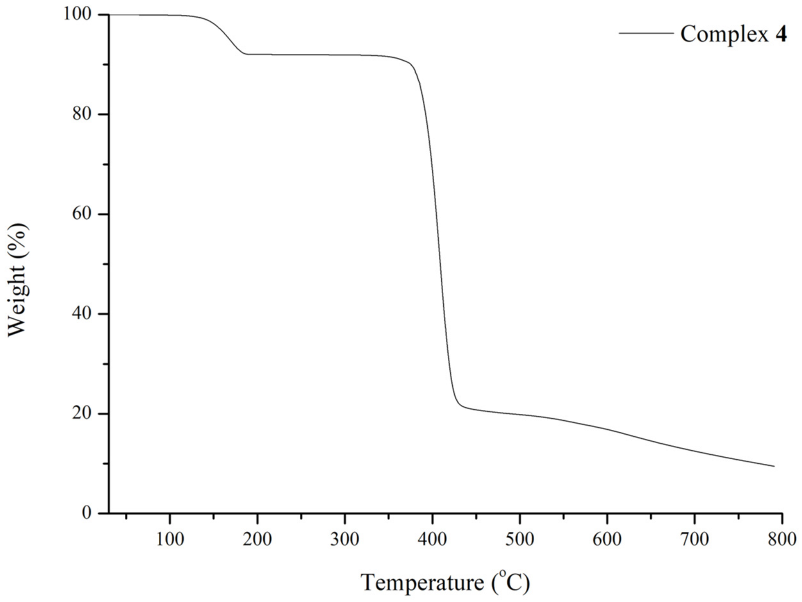

Figure S12. TGA curve of complex **5**.

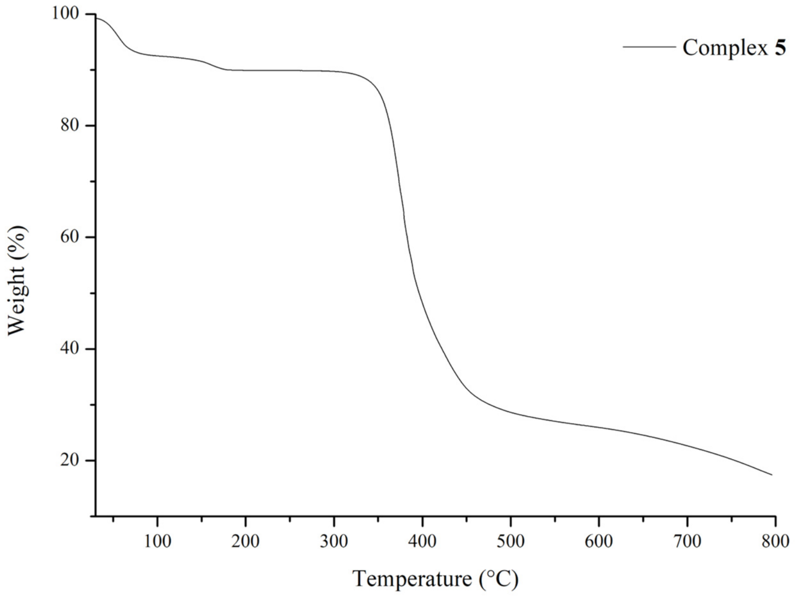

Figure S13. TGA curve of complex **6**.

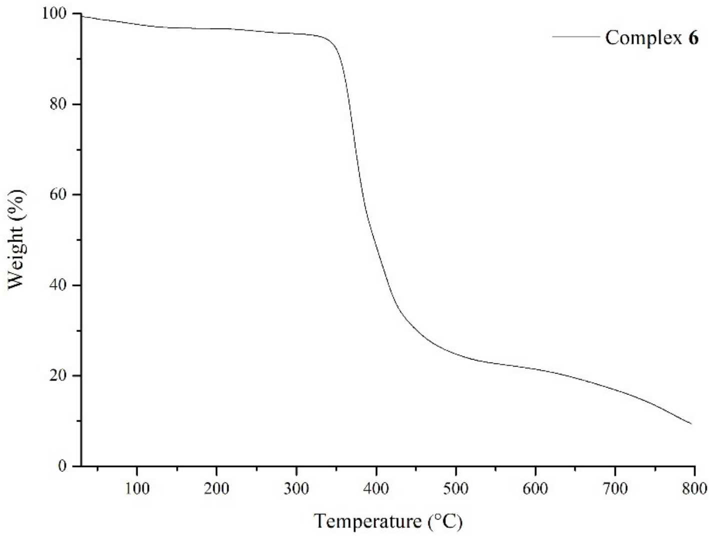

Figure S14. TGA curve of complex **7**.

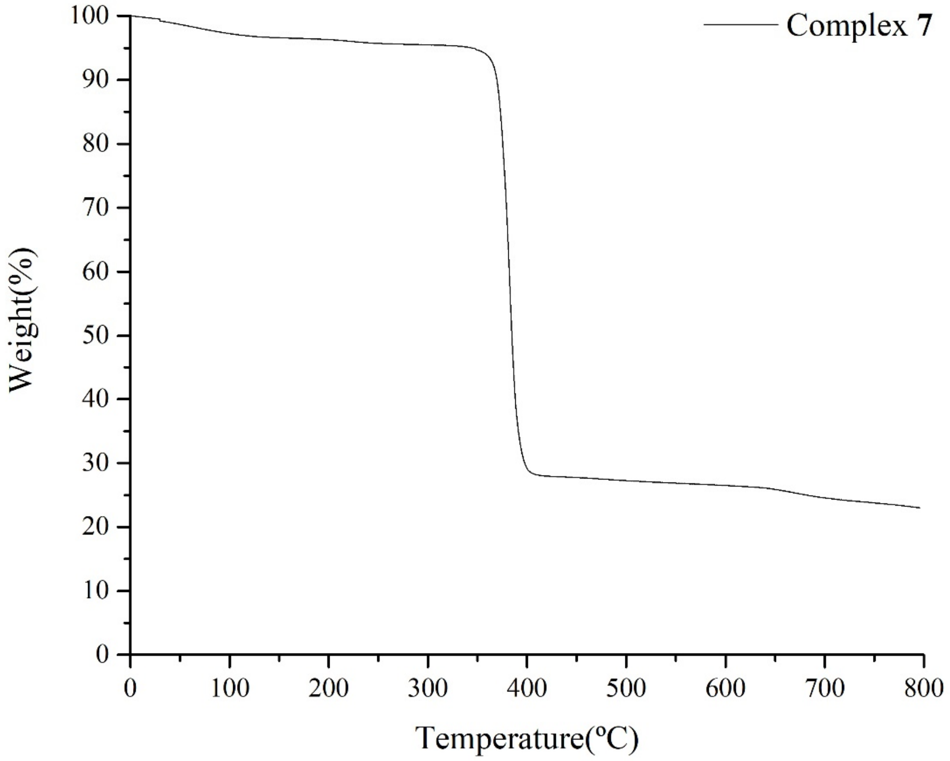

Figure S15. Solid-state luminescence spectrum of 1,3-H_2BDC.

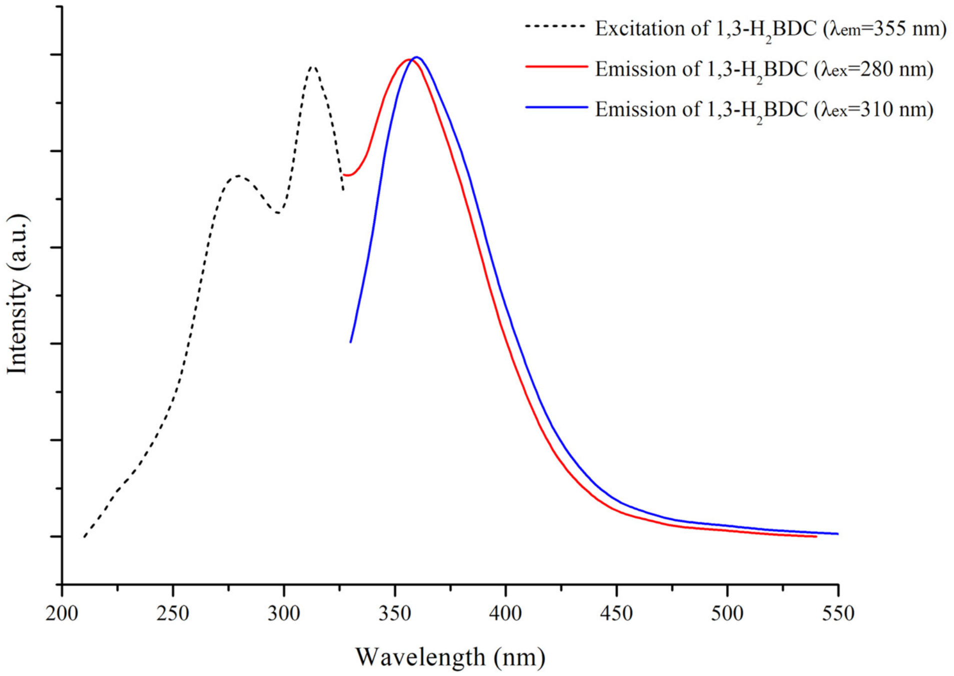

Figure S16. Solid-state luminescence spectrum of 1,4-H_2BDC.

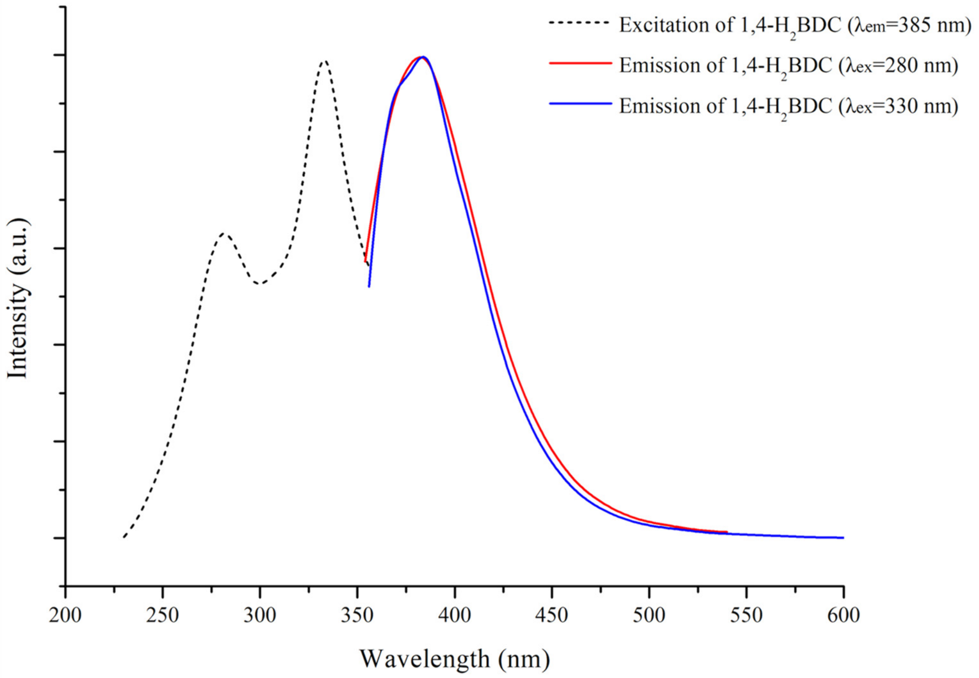

Figure S17. Solid-state luminescence spectrum of **L**.

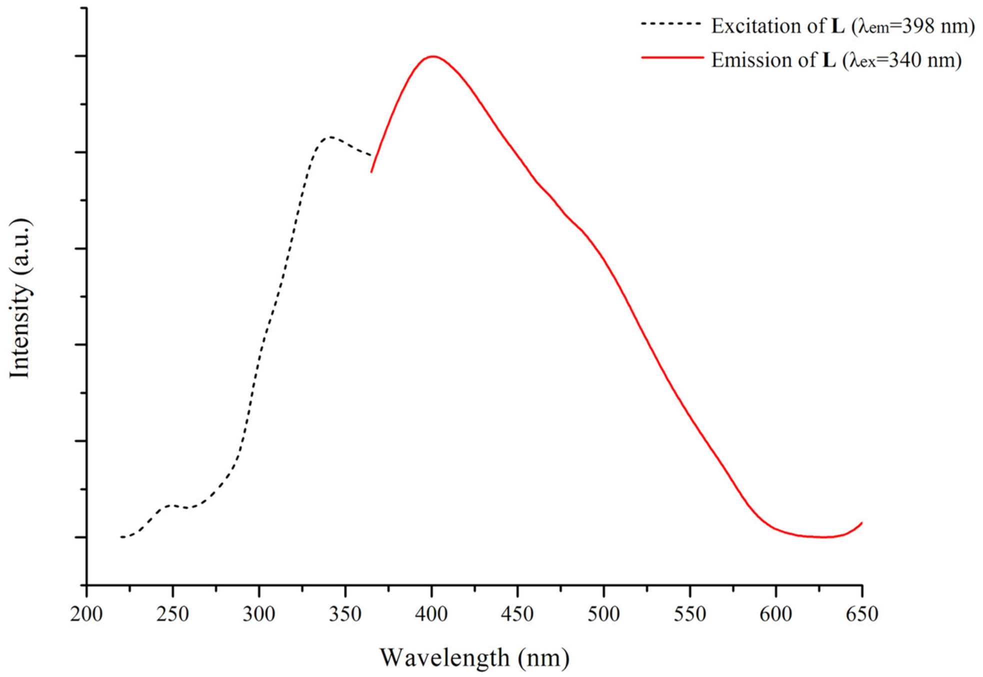

Figure S18. Solid-state luminescence spectrum of complex **1**.

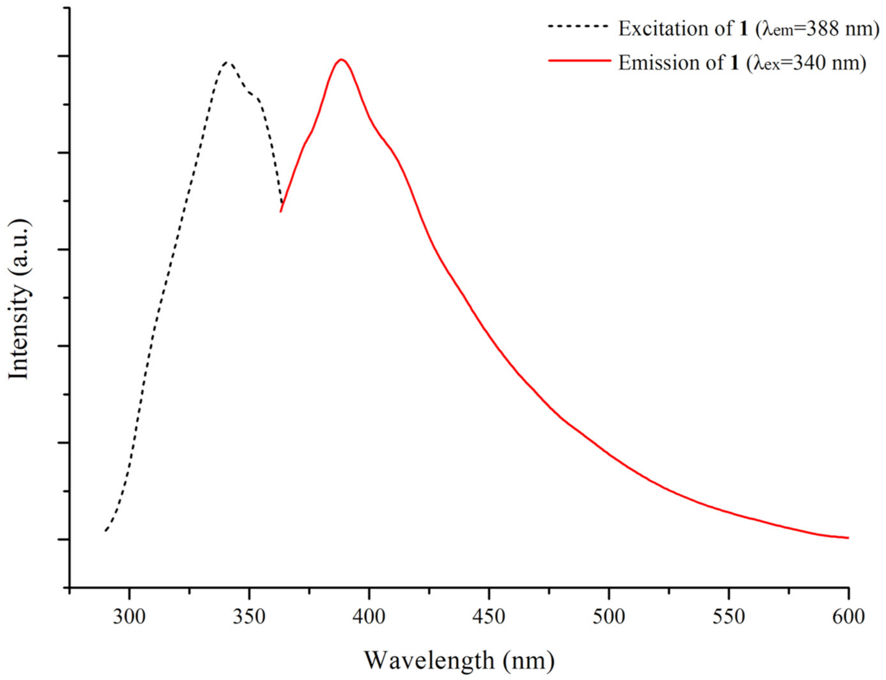

Figure S19. Solid-state luminescence spectrum of complex **2**.

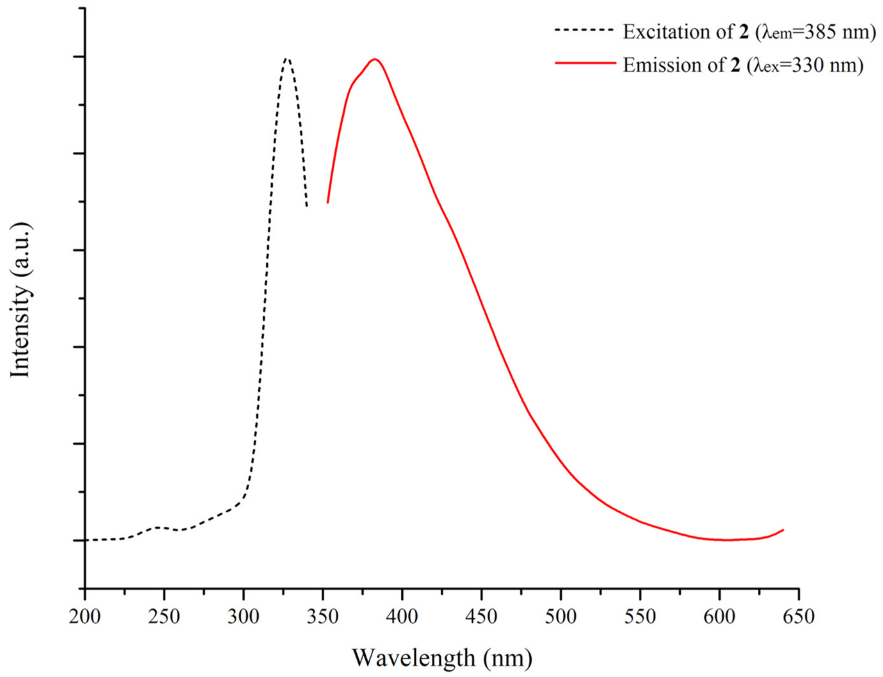

Figure S20. Solid-state luminescence spectrum of complex **5**.

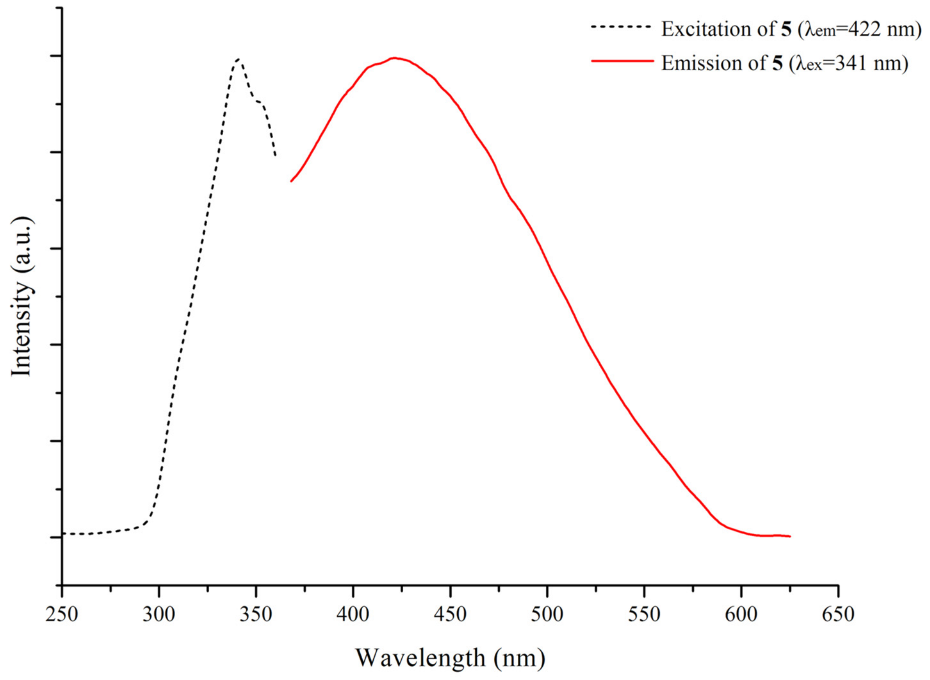

Figure S21. Solid-state luminescence spectrum of complex **6**.

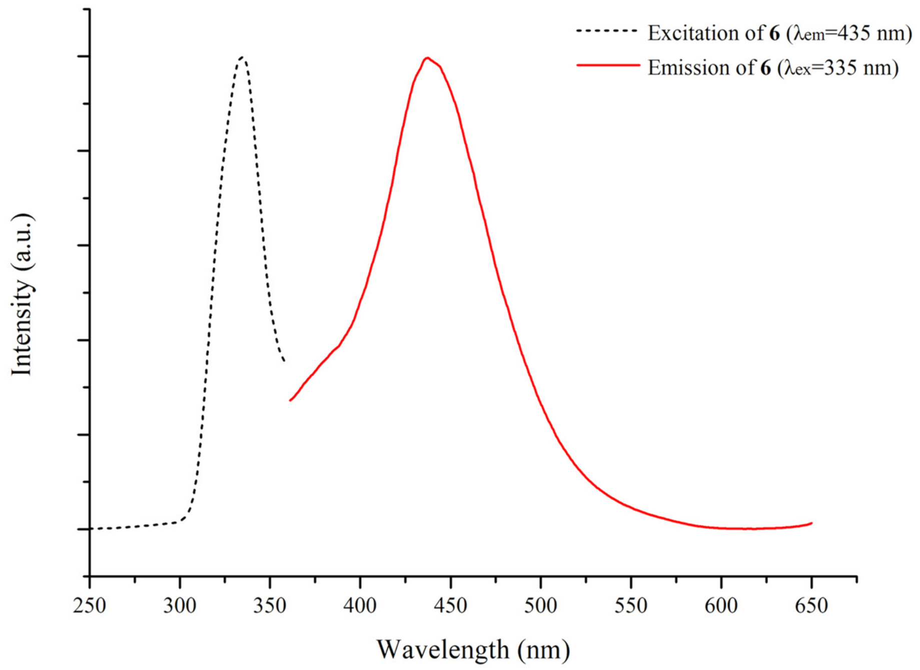

Figure S22. Solid-state luminescence spectrum of complex **7**.

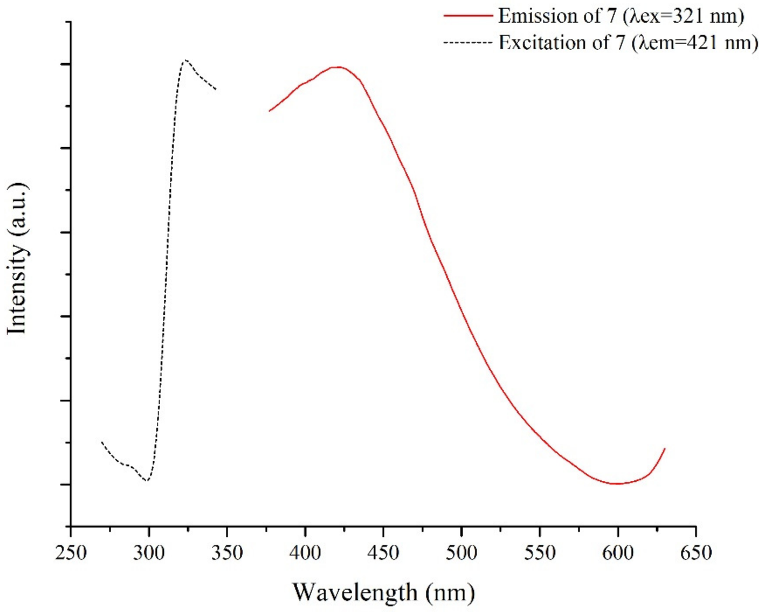

Figure S23. Luminescence intensities of **1** in first round upon immersion in the aqueous solutions of different metal ions. (a) Emission spectrum and (b) column diagram.

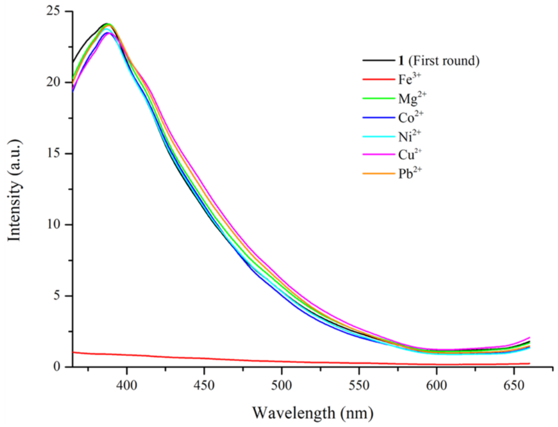

(a)

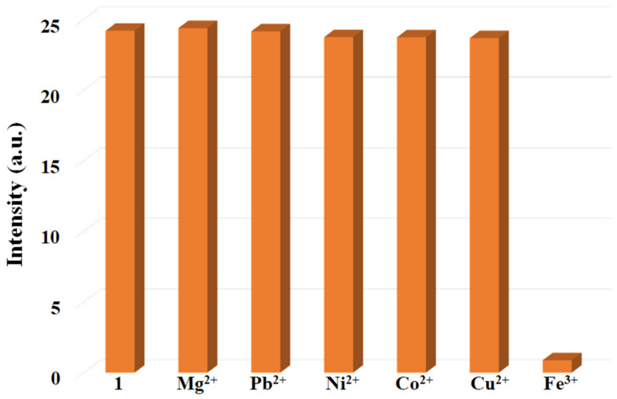

(b)

Figure S24. Luminescence intensities of **1** in second round upon immersion in the aqueous solutions of different metal ions. (a) Emission spectrum and (b) column diagram.

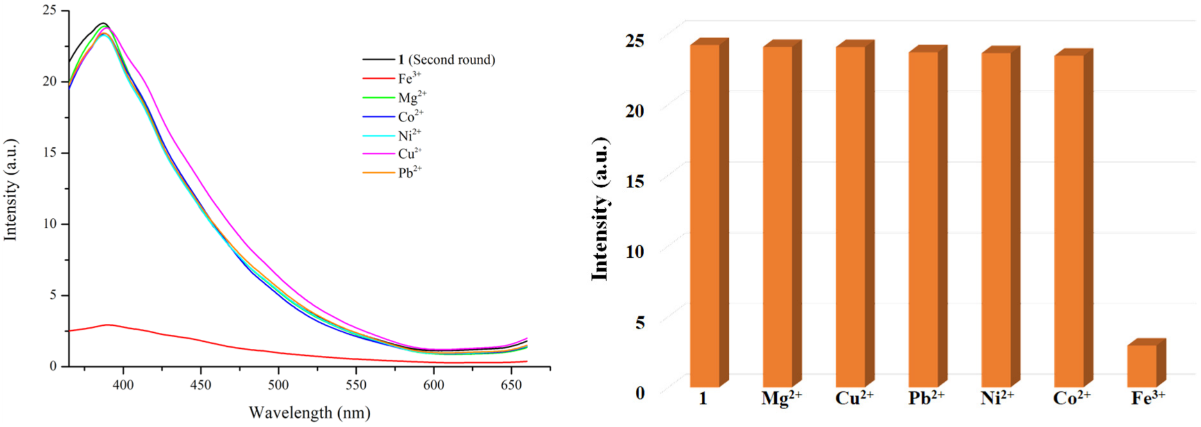

(a) (b)

Figure S25. Luminescence intensities of **1** in third round upon immersion in the aqueous solutions of different metal ions. (a) Emission spectrum and (b) column diagram.

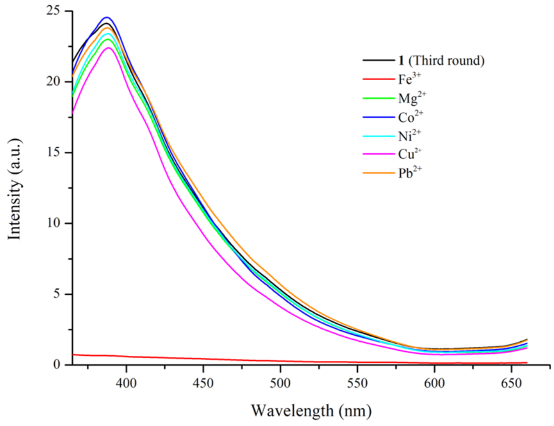

(a)

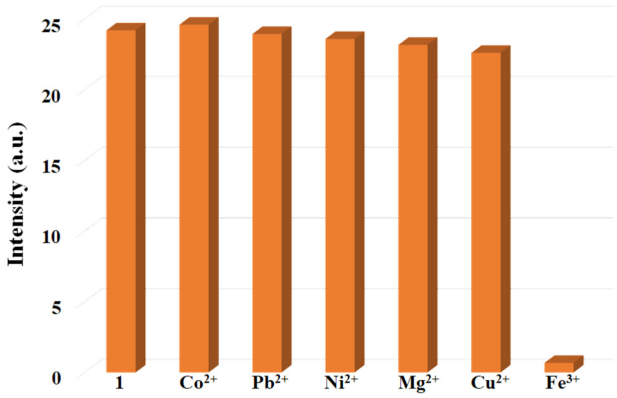

(b)

Figure S26. Luminescence intensities of **5** in first round upon immersion in the aqueous solutions of different metal ions. (a)Emission spectrum and (b)column diagram.

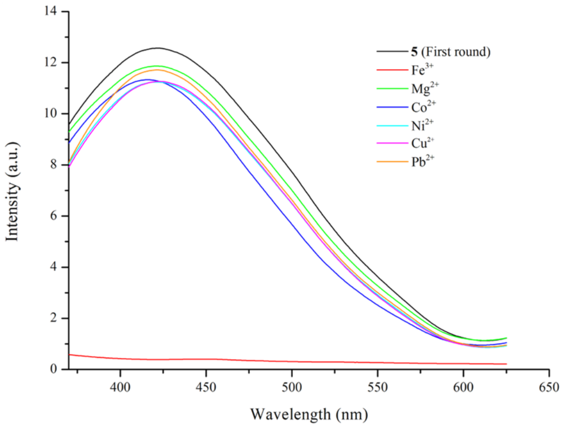

(a)

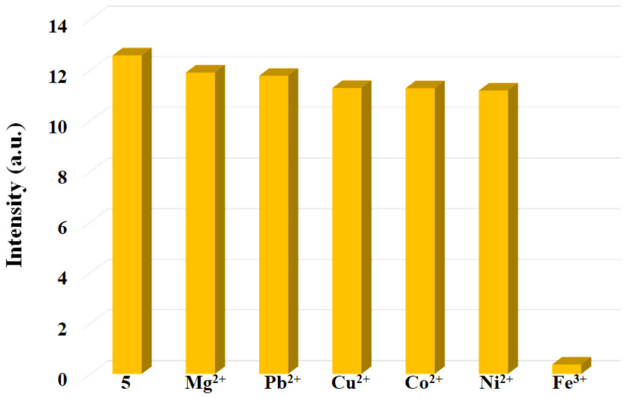

(b)

Figure S27. Luminescence intensities of **5** in second round upon immersion in the aqueous solutions of different metal ions. (a)Emission spectrum and (b)column diagram.

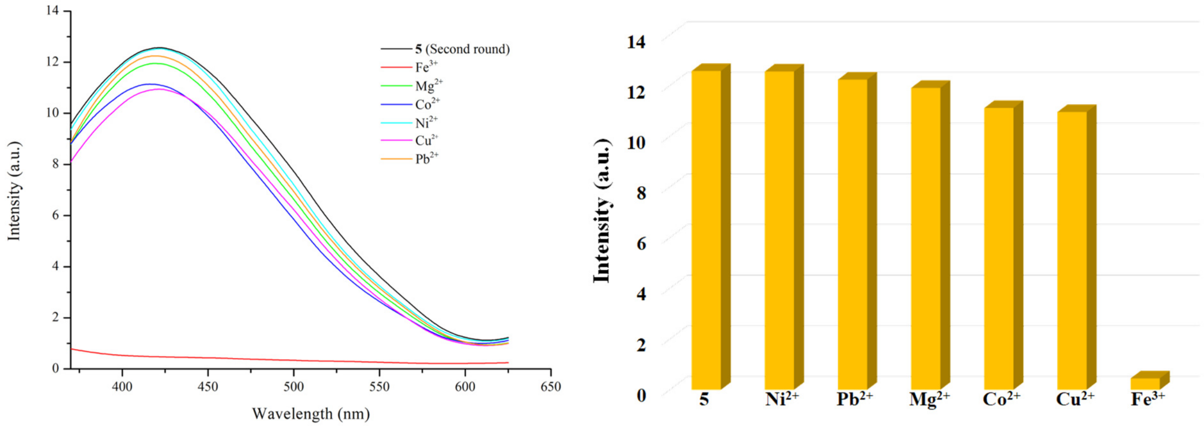

(a) (b)

Figure S28. Luminescence intensities of **5** in third round upon immersion in the aqueous solutions of different metal ions. (a)Emission spectrum and (b)column diagram.

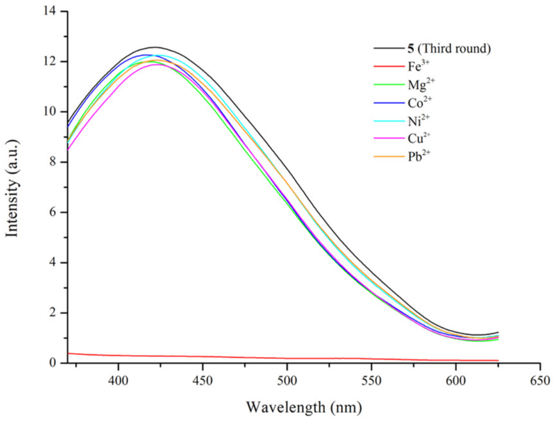

(a)

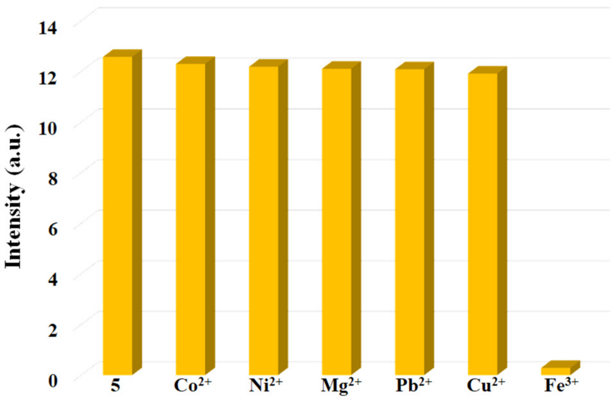

(b)

Figure S29. Luminescence intensities of first round of **1** upon immersion in the aqueous solutions of different metal ions.

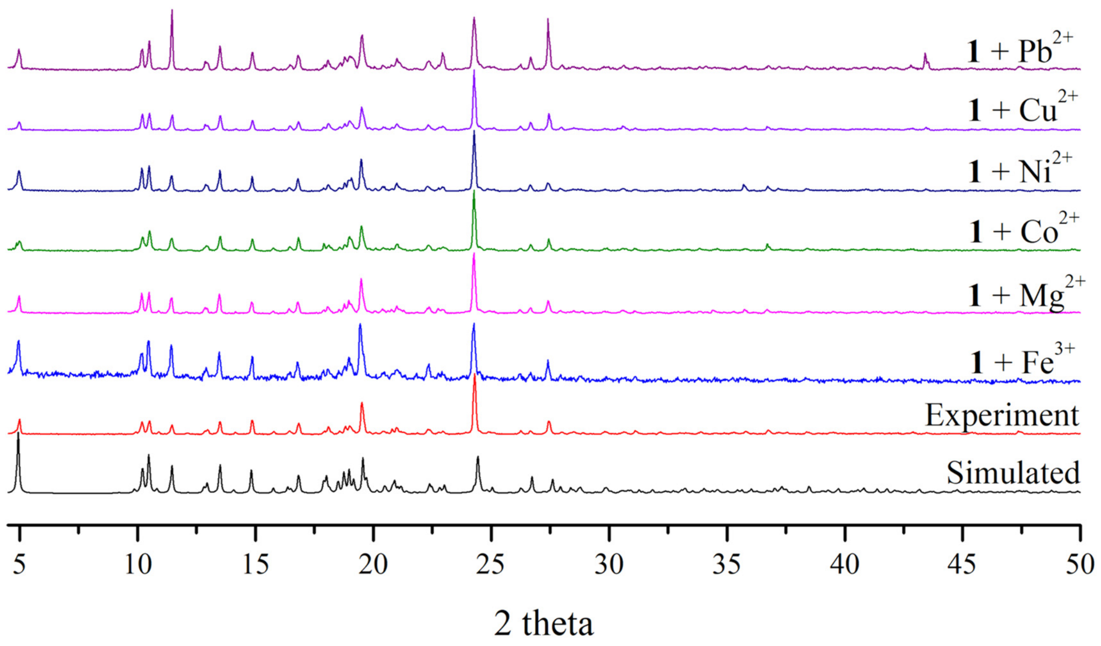

Figure S30. Luminescence intensities of second round of **1** upon immersion in the aqueous solutions of different metal ions.

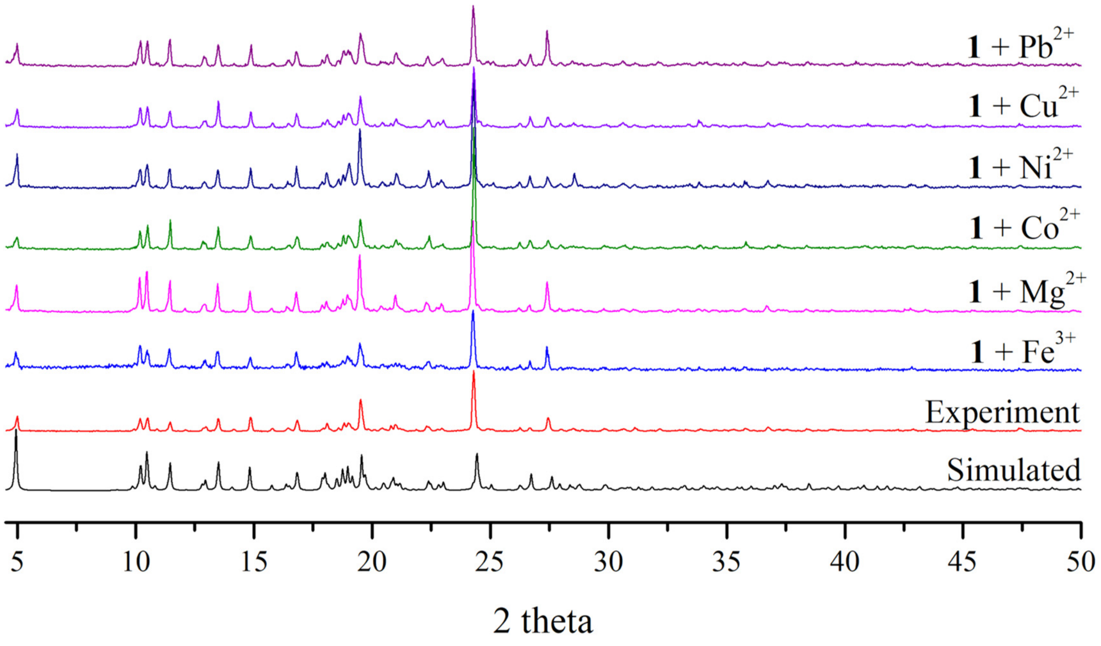

Figure S31. Luminescence intensities of third round of **1** upon immersion in the aqueous solutions of different metal ions.

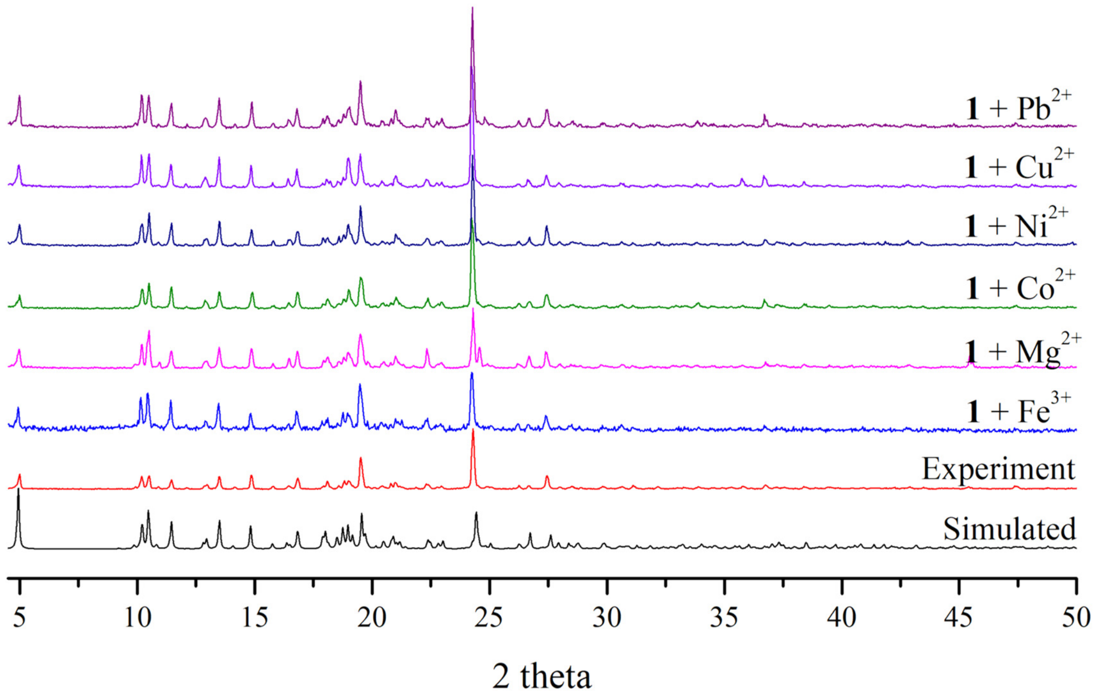

Figure S32. Emission intensities of complex **1** immersed in Fe^{3+} with various concentrations.

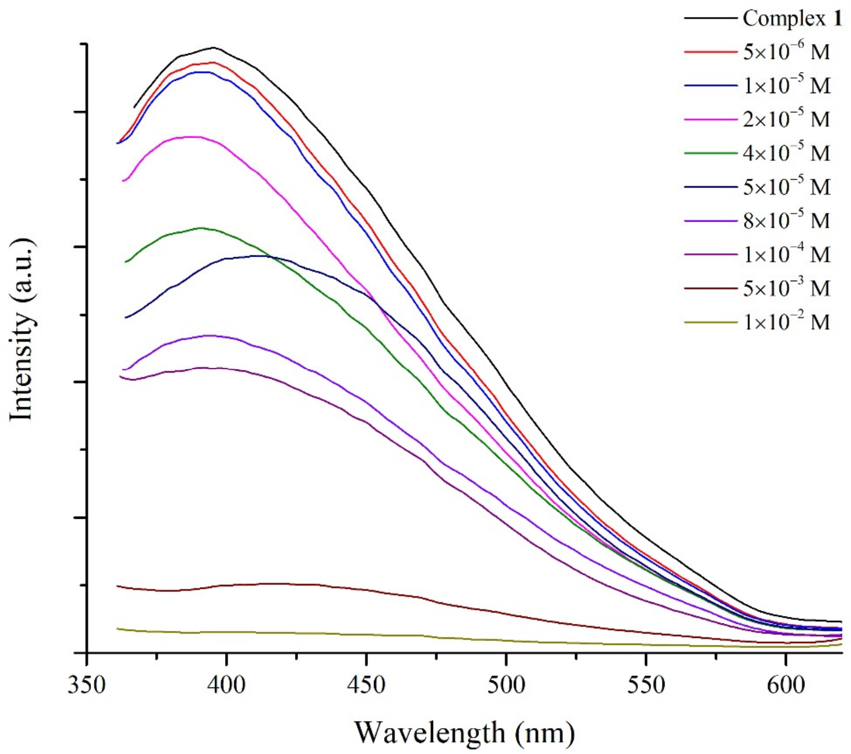

Figure S33. PXRD patterns of complex **1** in Fe^{3+} solution for five cycles.

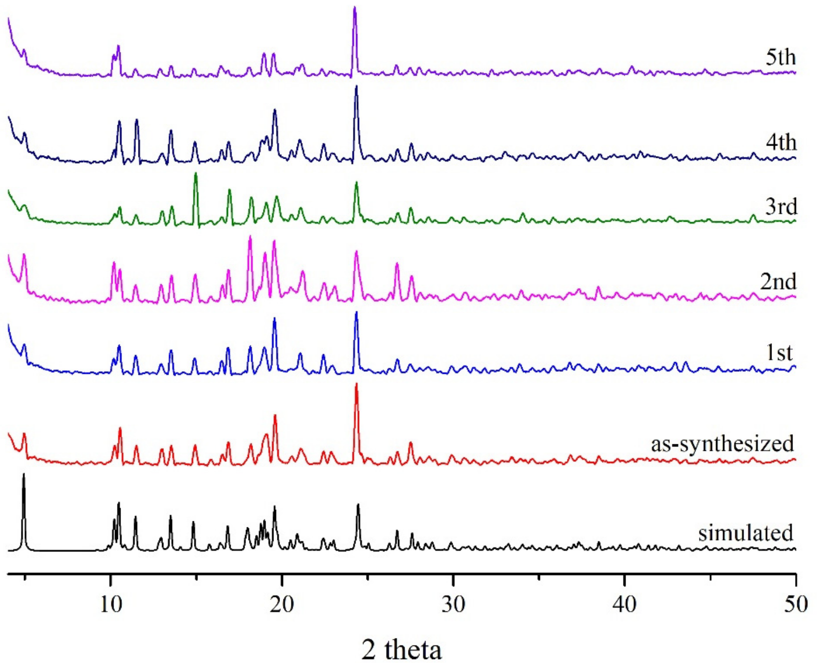

Supplement: Supplementary file 1 [file molecules-30-03283-s001.zip › molecules-3793307-supplementary.pdf]
